# Norepinephrine Controls Both Torpor Initiation and Emergence via Distinct Mechanisms in the Mouse

**DOI:** 10.1371/journal.pone.0004038

**Published:** 2008-12-24

**Authors:** Steven J. Swoap, David Weinshenker

**Affiliations:** 1 Department of Biology, Williams College, Williamstown, Massachusetts, United States of America; 2 Department of Human Genetics, Emory University School of Medicine, Atlanta, Georgia, United States of America; University of Parma, Italy

## Abstract

Some mammals, including laboratory mice, enter torpor in response to food deprivation, and leptin can attenuate these bouts of torpor. We previously showed that dopamine β-hydroxylase knockout (*Dbh* −/−) mice, which lack norepinephrine (NE), do not reduce circulating leptin upon fasting nor do they enter torpor. To test whether the onset of torpor in mice during a fast requires a NE-mediated reduction in circulating leptin, double mutant mice deficient in both leptin (*ob/ob*) and DBH (DBL MUT) were generated. Upon fasting, control and *ob/ob* mice entered torpor as assessed by telemetric core T_b_ acquisition. While fasting failed to induce torpor in *Dbh −/−* mice, leptin deficiency bypassed the requirement for NE, as DBL MUT mice readily entered torpor upon fasting. These data indicate that sympathetic activation of white fat and suppression of leptin is required for the onset of torpor in the mouse. Emergence from torpor was severely retarded in DBL MUT mice, revealing a novel, leptin-independent role for NE in torpor recovery. This phenotype was mimicked by administration of a β_3_ adrenergic receptor antagonist to control mice during a torpor bout. Hence, NE signaling via β_3_ adrenergic receptors presumably in brown fat is the first neurotransmitter-receptor system identified that is required for normal recovery from torpor.

## Introduction

Under acute food deprivation and cool ambient temperature (T_a_), laboratory mice allow their body temperature to fall to near ambient temperature, with a minimum body temperature around 20°C and a concomitant reduction in metabolic rate [1]–[6]. This heterothermic response contrasts with true hibernators, like ground squirrels, that can achieve a minimum body temperature at or below freezing for weeks at a time [7]. Common to both daily heterotherms like the mouse and hibernators is the endogenous production of heat to facilitate exit from torpor. Heat can be generated via general activity (i.e. voluntary muscle contractions), shivering (i.e. involuntary muscle contractions), and through futile cycling of ions, such as protons or calcium. In small mammals such as mice, an important source of rapid heat production comes from brown fat thermogenesis, which involves uncoupling the synthesis of ATP from the proton gradient [8]. In general, heat production, including brown fat thermogenesis, is primarily under the control of the sympathetic nervous system (SNS) in mammals. The SNS is also critical for preventing heat loss in mice via vasoconstriction of the tail vasculature [9].

Epinephrine (Epi) and norepinephrine (NE) are the primary neurotransmitters for the SNS. Using dopamine β-hydroxylase knockout (*Dbh −/−*) mice that lack both Epi and NE, we wished to test the hypothesis that these neurotransmitters are required for the thermogenic events required for exit from torpor. However, we could not test this hypothesis when we were surprised to learn that *Dbh −/−* mice failed to enter torpor under inducing conditions [10]. Both brown adipose tissue (BAT) and white adipose tissue (WAT) are innervated by the SNS [11]–[13]. Pharmacological evidence suggested that the lack of sympathetic activation of WAT, mediated by the β3-adrenergic receptor, and subsequent failure to lower circulating leptin levels prevented the *Dbh −/−* mice from entering torpor [10]. Indeed, administration of leptin attenuates torpor bouts in daily heterotherms [14], [15], although low circulating leptin levels are not the only signal for torpor entry [16].

To test whether *Dbh −/−* mice do not enter torpor upon fasting because of their failure to suppress circulating leptin levels, we bred *Dbh −/−* mice onto a leptin-deficient (*ob/ob*) background to produce double knockout (DBL MUT) mice. We reasoned that if the lack of torpor in *Dbh −/−* mice was a result of hyperleptinemia, the DBL MUT mice would enter torpor bouts upon fasting. Our results show that these mice do in fact enter daily torpor upon fasting. Hence, we used these mice to test our original hypothesis that exit from a torpor bout requires sympathetic release of NE for endogenous heat production. Our data identify NE and β3-adrenergic receptors as the first neurotransmitter-receptor system required for the normal emergence from torpor in the mouse.

## Materials and Methods

### Breeding scheme for mice

*Dbh −/−* males were bred to *ob/+* heterozygous females to produce *Dbh+/−*, *ob/+* offspring. These double heterozygotes were bred to each other to produce the final breeder males (*Dbh −/−*, *ob/+*) and females (*Dbh +/−*, *ob/+*). These breeders were crossed to generate the 4 genotypes tested in the experiments: control (*Dbh +/−*, *ob/+ or +/+*), NE-deficient (*Dbh −/−*, *ob/+* or *+/+*), leptin-deficient (*Dbh +/−*, *ob/ob*), or both NE- and leptin-deficient (*Dbh −/−*, *ob/ob*), named DBL MUT. Pregnant dams carrying potential *Dbh −/−* fetuses were given the adrenergic receptor agonists isoproterenol and phenylephrine from E9.5–E14.5 and the synthetic NE precursor L-3,4-dihydroxyphenylserine (DOPS) from E14.5-birth in their drinking water to rescue the embryonic lethality associated with the homozygous *Dbh −/−* mutation, as described [17]. *Dbh −/−*, *ob/+* breeder males were given daily DOPS injections to rescue the male fertility deficit associated with the *Dbh −/−* mutation [17]. All genotypes were confirmed by PCR. *Dbh +/−* mice were used as controls because they have normal catecholamine levels and are indistinguishable from wild-type mice for all previously tested phenotypes, including torpor [10], [17]. No differences were observed between *ob/+* and *+/+* mice, and results were combined. All mice were born and reared in a specific pathogen-free facility at Emory University with a 12 h light/dark cycle (lights on - 7 am; lights off - 7 pm). Food and water were available *ad libitum* (except during torpor experiments, as described below). All mice were shipped to Williams College for physiological assessment. Studies were approved by each of the local IACUCs and guidelines for animal husbandry of both institutions were followed.

### Reagents

The β3 adrenergic receptor antagonist, SR59230A, was purchased from Sigma-Aldrich. DOPS was a gift from Dainippon-Sumitomo Pharma, Co., Ltd. (Osaka, Japan).

### Implantation of temperature telemeters

Male and female mice aged 6 months were used in all experiments. No gender differences were found, and results were combined. Mice (n = 6 for control, n = 16 for *ob/ob*, n = 5 for *Dbh* −/−, n = 6 for DBL MUT) were implanted with temperature telemeters as described previously [10]. The mice were maintained on a heating pad for 48 hours following the surgery, and then housed individually at 28–30°C for 10 days to allow time for recovery.

### Temperature data collection and analysis

Data from the temperature telemeters were recorded at 500 Hz, for one second, once per minute for 23 hours using Data Sciences Int. acquisition software. To calculate maximum rates of temperature gain and temperature loss, a 30 minute sliding window was used to calculate the rate of temperature change. The maximums and minimums of this data series were then calculated.

### Experimental protocol

After the 10 day recovery period from surgery, mice were moved to a temperature-controlled cage (±0.25°C) at 21°C and held there for two days. On day one, the mice had free access to food and water. On day two, the mice were fasted at the onset of the dark cycle, but had free access to water. In one experiment, mice were injected with SR59230A, a potent β3 adrenergic receptor antagonist [18]. SR59230A was dissolved in DMSO, and diluted in sterile phosphate-buffered saline (PBS) to a concentration of 2.5 mg/ml. DMSO was at a final concentration of 3%. 0.2 ml of either SR59230A or vehicle (3% DMSO in PBS) was injected subcutaneously between the scapula in mice in fasting-induced torpor at a T_b_ of ∼25°C.

### Statistics

GraphPad Prism 4.0c for Macintosh was used for all statistical tests. All results are reported as means±SE. One-way ANOVAs, followed by post-hoc Newman-Keuls tests, were used to determine statistical significance for each parameter. A student t-test was used to determine statistical significance for maximum rate of temperature elevation in the β3 antagonist experiment. Significance levels of *P*<0.05 were considered significant.

## Results

### Leptin deficiency restores fasting-induced torpor in *Dbh −/−* mice

**Figure 1:**
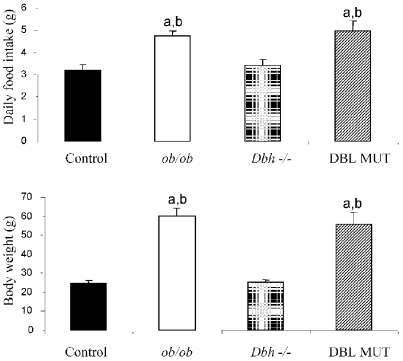
Epinephrine and norepinephrine play no role in the hyperphagia and obesity associated with a lack of leptin. Before the mice of each of the four genotypes (control, *Dbh −/−*, *ob/ob*, and DBL MUT) were implanted with temperature telemeters, the mice were weighed ∼6 months of age. Food intake was measured for one week while housed at an ambient temperature of 28°C. DBL MUT and *ob/ob* mice were both hyperphagic [F(26,3) = 8.702, P<0.0005] and obese [F(27,3) = 37.46, P<0.0001] relative to their counterparts. These data suggest that Epi and NE play little role in food consumptive behavior and associated obesity due to the lack of leptin. a : p<0.05 vs. control. b : p<0.05 vs. *Dbh −/−*.

**Figure 2:**
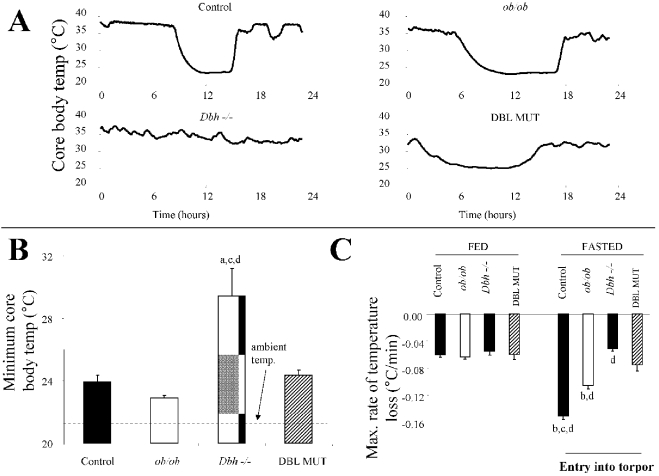
Leptin deficiency restores torpor in *Dbh −/−* mice. (A) Typical tracings of core body temperature for a 24 hour fasting period are shown for control, *ob/ob*, *Dbh* −/− and DBL MUT mice. Fasting was initiated at the beginning of the 12 hour dark cycle, which started at time 0. *Dbh* −/− mice clearly do not enter torpor with fasting, whereas the DBL MUT mice enter torpor almost immediately after initiation of the fast. (B) The minimum core body temperature during the 24 hour fast was calculated. An ambient temperature of 21°C during the fast is shown. [F(32,3) = 26.71, P<0.0001]. a : p<0.05 vs. control. c : p<0.05 vs. *ob/ob*. d: p<0.05 vs. DBL MUT. (C) The maximum rates of temperature decline were calculated for all four genotypes during both the fed period, and during a fast over a sliding 30 minute window using the first derivative of core body temperature curves. [F(32,3) = 29.11, P<0.0001]. b : p<0.05 vs. *Dbh −/−*. c : p<0.05 vs. *ob/ob*. d: p<0.05 vs. DBL MUT.

Mice that are deficient in both leptin and DBH are viable following prenatal NE and Epi replacement. These DBL MUT mice are hyperphagic and obese to the same extent as *ob/ob* mice (Fig. 1). DBL MUT mice are somewhat hypothermic while fed at a T_a_ of 21°C, with a core T_b_ ∼1–2°C below that of littermate controls (Table). Mice of all four genotypes (control, *Dbh −/−*, *ob/ob*, and DBL MUT) were placed under conditions conducive for torpor (fasted at a T_a_ of 21°C). Control and *ob/ob* mice entered torpor bouts as assessed by core T_b_ measurements (Fig. 2A). As we have shown previously [10], *Dbh−/−* mice did not enter torpor upon fasting (Fig. 2A). DBL MUT mice, however, experienced a significant bout of hypothermia when fasted that displayed many characteristics of torpor. The depth of hypothermia was similar between control, *ob/ob*, and the DBL MUT mice, ∼3°C above ambient temperature (Fig. 2B). The rate of entry into torpor was slower for *ob/ob* mice than control mice (Fig. 2C), likely due to the larger size of the *ob/ob* mice. The rate of entry into torpor for the DBL MUT was intermediate between the similar-sized *ob/ob* mouse and the *Dbh −/−* mouse, which did not enter torpor (Fig. 2C). DBL MUT mice entered torpor almost immediately upon fasting (see Fig. 2A) and reached their maximum rate of T_b_ decline within 1.8±0.3 hours of fasting, whereas *ob/ob* and control mice reached their maximum rate of T_b_ decline after 6.0±0.4 hours and 7.5±0.6 hours from the onset of the fast, respectively.

**Table 1:** Core body temperatures (°C) while fed and housed at 21°C.

	Control	*ob/ob*	*Dbh −/−*	DBL MUT
Maximum T_b_	38.4±0.1	36.8±0.2^a^ ^c^	37.9±0.3^b^ ^c^	35.7±0.7^a^
Average T_b_	36.4±0.2	35.0±0.2^a^ ^c^	35.4±0.4^c^	33.7±0.7^a^
Minimum T_b_	34.5±0.4	32.5±0.3^a^	33.3±0.7	31.9±0.9^a^

a p<0.05 vs. control.

b p<0.05 vs. *ob/ob*.

c p<0.05 vs. DBL MUT.

**Max temp**.

F(29,3) = 13.09, P<0.0001.

**Average temp**.

F(29,3) = 9.023, P = 0.0003.

**Min temp**.

F(29,3) = 4.241, P = 0.0144.

### Recovery from torpor is impaired in DBL MUT mice

**Figure 3:**
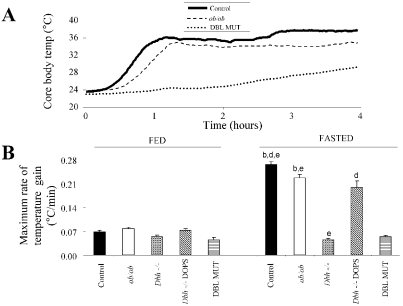
Emergence from torpor is retarded in DBL MUT mice. (A) Typical tracings from control, *ob/ob*, and DBL MUT mice during emergence from torpor. Both control and *ob/ob* mice exit torpor within 45 minutes of initiation, whereas emergence from torpor in this DBL MUT mouse was much slower. (B) The maximum rate of temperature gain over a 30 minute window (a measure of emergence from torpor) was calculated during both the fed state and fasted state based off the first derivative of core body temperature curves. Rapid emergence from torpor was seen in control, *ob/ob*, and *Dbh* −/− mice treated with DOPS to acutely replace NE. Data for “*Dbh* −/−+DOPS” groups was reanalyzed from ref. [10]. [F(39,4) = 42.68, P<0.0001]. b : p<0.05 vs. *Dbh −/−*. c : p<0.05 vs. *ob/ob*. d: p<0.05 vs. DBL MUT. e : p<0.05 vs. *Dbh −/−*+DOPS.

The ability of the DBL MUT mice to enter torpor allowed for the examination of NE deficiency on torpor recovery. The duration of time with a core T_b_ below 28°C was significantly greater (p<0.05) in DBL MUT mice (52±8% of their day) than in *ob/ob* mice (33±3%), which in turn was significantly greater (p<0.05) than that of control mice (21±3%) [F(33,3) = 30.42, P<0.0001]. These genotype differences in time spent torpid were in large part driven by the rate of T_b_ gain during torpor recovery. Control and *ob/ob* mice emerged from torpor within 30–45 minutes of initiation of recovery, whereas the DBL MUT mice were still well below their feeding T_b_ after 4 hours (Fig. 3A). Control mice exited torpor at a maximum rate of temperature gain of 0.26°C per minute, whereas temperature gain was moderately slower in *ob/ob* mice (0.23°C per minute; Fig. 3B), likely due to their increased mass. In contrast, the DBL MUT mice emerged from torpor at a rate of only 0.05°C per minute, which was not different from the maximum rate of temperature gain during the feeding period (Fig. 3B). These data indicated that NE is required for torpor recovery as well as torpor initiation. To further examine the role of NE in exit from torpor, we reanalyzed published data from single mutant *Dbh −/−* mice (n = 12) that were administered L-3,4-dihydroxyphenlserine (DOPS), a synthetic NE precursor that transiently restores NE and torpor entry to these mice [10]. We found that treatment with DOPS also restored a normal torpor recovery rate in *Dbh −/−* mice; arousal from torpor in DOPS-treated *Dbh −/−* mice was at a rate similar to that of wild-type mice (Fig. 3B).

### A β3 antagonist impairs recovery from torpor in control mice

**Figure 4:**
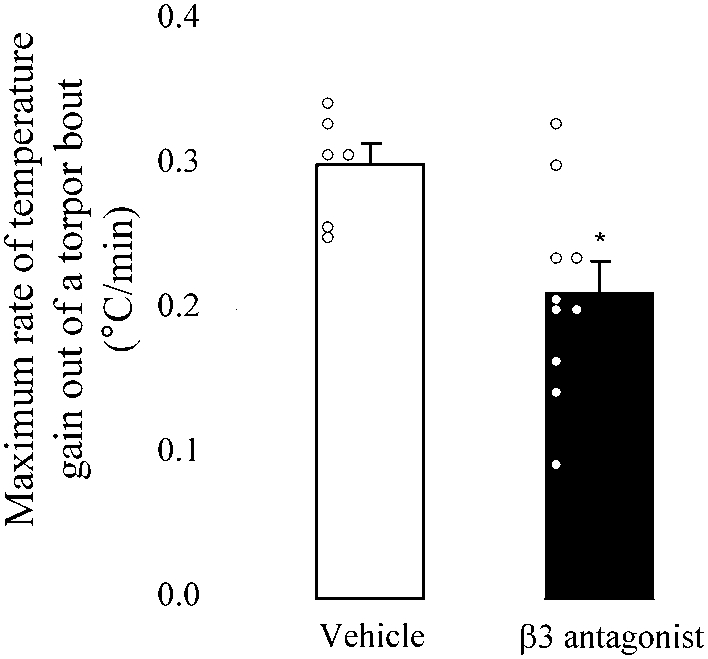
Administration of a β3 antagonist slows rate of emergence from torpor. 16 control mice were fasted at the onset of the dark cycle. Once in torpor, mice were either injected subcutaneously with 2 mg of the β3 antagonist, SR59230A, or vehicle. The maximum rate of temperature elevation was calculated as in Figure 2. The rates for individual mice in each group are shown. [t-test: t = 2.844, df = 14, p = 0.013 vs. vehicle injection].

If NE activation of the β3 adrenergic receptor on brown fat is required for rapid thermogenesis and recovery from torpor, then administration of a β3 antagonist should blunt the rate of recovery from a bout of torpor. To test this prediction, control mice (n = 16) were fasted to induce a bout of torpor. When their T_b_ was ∼25°C, the mice were administered either vehicle (n = 6) or the β3 antagonist SR59230A (n = 10) via a subcutaneous injection in the vicinity of the interscapular brown fat pads. Handling of the mice for subcutaneous injection caused immediate arousal from the torpid state, but the rate of arousal following β3 antagonist administration was blunted (70% of the rate following vehicle administration) (Fig. 4). These data, combined with the slow recovery of torpor in DBL MUT mice, demonstrate the requirement for activation of the β3 adrenergic receptor by NE for normal emergence from torpor.

## Discussion

The breeding of DBH deficiency onto an *ob/ob* background has allowed for the testing of two separate hypotheses concerning noradrenergic control of entrance and emergence from torpor. First, we had found previously that *Dbh −/−* mice do not enter torpor under conditions where wild-type mice do [10], namely a 24 hour fast at a typical housing temperature (21°C). This phenotype was rescued in *Dbh −/−* mice by administration of a β3-adrenergic receptor agonist and mimicked in control mice by a β3 antagonist [10]. The correlation between the lack of torpor in *Dbh −/−* mice and the failure to suppress leptin levels during a fast led us to postulate that it was the inability to engage the SNS, activate β3 receptors and suppress leptin secretion from white adipose tissue during a fast that kept these mice from entering torpor. Our second hypothesis was that emergence from torpor requires thermogenesis driven by sympathetic innervation of brown adipose tissue, an idea that could not be tested in *Dbh −/−* single mutant mice due to their inability to enter torpor.

**Figure 5:**
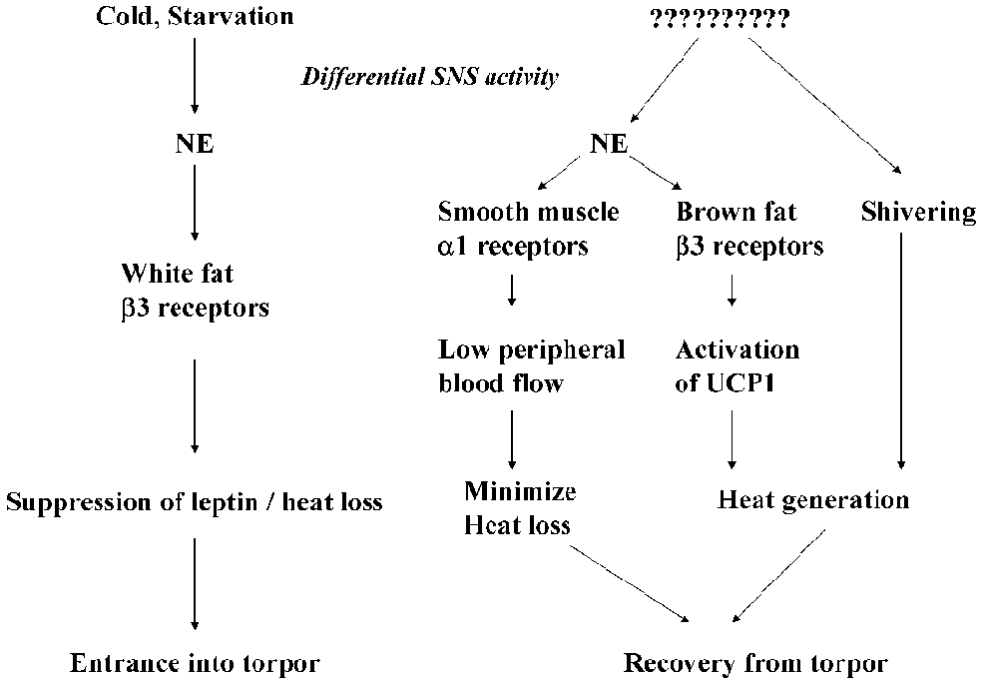
Hypothetical model of torpor induction and emergence in the mouse. During an acute food shortage, sympathetic discharge to white adipose tissue increases. Activation of the β3 adrenergic receptor on white adipose tissue results in a fall in circulating leptin which is required for entrance into torpor. At some point during the torpor bout, sympathetic discharge to β3 adrenergic receptors on brown fat activates existing UCP1 proteins resulting in heat production and ultimately exit from torpor. Decreased peripheral blood flow is also required to retain heat generated by brown fat, as well as shivering.

The data presented here support the first hypothesis, that elevated leptin levels prevent the *Dbh −/−* mouse from entering fasting-induced torpor. We found that leptin deficiency was epistatic to NE deficiency; in contrast to the slow and modest hypothermia observed in fasted *Dbh −/−* mice, DBL MUT mice were able to respond to a fast with a true torpor bout. Further, we have found in a preliminary experiment that exogenous replacement of leptin in a DBL MUT mouse prevents torpor and causes a reversion back to the *Dbh −/−* single mutant phenotype (unpublished observations). These results suggest that entrance into torpor requires the sequential SNS release of NE, activation of β3-adrenergic receptors on white adipocytes, and the suppression of leptin secretion (Figure 5, left pathway).

Because the DBL MUT mice were able to enter torpor, we could test our second hypothesis, that NE is required for exit from torpor. We found that while DBL MUT mice initiated torpor recovery at an appropriate time (∼5 hours after reaching minimum T_b_), the rate of temperature gain was severely retarded. The rate at which a mouse recovers from torpor will be dependent upon both heat production and heat loss. It appears that DBL MUT mice have a deficit in both heat production and heat retention, which would explain their slow arousal from torpor. The two primary sources of heat production available to small mammals are non-shivering thermogenesis (NST) and shivering. NST in mice is achieved predominantly through activation of uncoupling protein 1 (UCP1), which requires sympathetic release of NE and activation of β3-adrenergic receptors in brown adipose tissue [8], [19]. The thermogenic role of brown adipose tissue in hibernators has been well established; indeed, this tissue was initially named the “hibernation gland” [20]. Activity of UCP1 within brown adipose is blocked both during hibernation and during daily torpor and unblocked during arousal from both states with the resultant elevation of temperature within brown adipose [2], [21]–[23]. *Dbh −/−* mice have low basal UCP1 levels and cannot induce UCP1 in response to cold [9], and the data presented here indicate that the slow emergence from daily torpor in DBL MUT mice may be partially explained by their inability to activate UCP1 within brown fat. When control mice were treated with a β3 antagonist during torpor in an attempt to inhibit NE-induced thermogenesis from brown fat, rates of arousal from torpor were significantly slowed, although not as slow as the DBL MUT mice (Fig. 4). There are several possible explanations for this disparity. For example, there is a total genetic lack of NE/β3 signaling in *Dbh −/−* mice, whereas antagonist treatment results in partial pharmacological blockade in the presence of competition from the endogenous ligand. In addition, DBL MUT mice were undisturbed, while control mice were injected with antagonist, and handling itself precipitates rapid recovery from torpor. Finally, SR59230A may have impacted lipolysis from white fat to influence the recovery rate from torpor, or had other effects in addition to β3 receptor antagonism that influenced torpor recovery.

Shivering is also a major component of heat generation in small mammals. Although *Dbh −/−* mice are cold sensitive due to lack of brown fat thermogenesis, they can shiver normally [9]. Both *ob/ob* mice, which have very little UCP1 [24], and UCP1 −/− mice are cold sensitive if immediately exposed to 4°C, but can tolerate this temperature if they undergo a cold adaptation protocol which allows time to increase shivering capacity [25], [26]. Indeed, *ob/ob* mice as well as many small marsupials exhibit rapid recovery from torpor despite having very little brown fat [27]–[29]. Although it remains to be determined, the DBL MUT mice likely have the ability to shiver. Thus, the lack of brown fat thermogenesis from the *Dbh −/−* mutation likely contributes to a severe impairment in heat generation in the DBL MUT mice.

The other side of the heat balance equation that allows for rapid arousal from a low body temperature is retention of heat generated. *Dbh −/−* and DBL MUT mice lack NE, the neurotransmitter not only required for sympathetic activation of fat, but also for α1-adrenergic receptor-mediated vasoconstriction of smooth muscle beds. Therefore, *Dbh −/−* mice are impaired in their ability to reduce blood flow to the periphery [9]. The hypothesis that *Dbh −/−* mice lose more heat through the periphery than control mice is supported by the observation that these mice eat similar amounts of food (Fig. 1) relative to their controls (*Dbh −/−* compared to control and DBL MUT compared to *ob/ob*), yet have a cooler body temperature (Table). However, the cooler body temperature of DBL MUT mice may be a reflection of energy storage at the expense of heat generation through metabolism. On the whole, it appears that heat generated by shivering in a mouse deficient in DBH is only partially retained, which combined with the deficit in UCP1 induction, further explains why the DBL MUT mice emerge from torpor so slowly.

We propose a model of fasting-induced torpor in a daily heterotherm that has differential sympathetic activation of adipose tissue at its core (Fig. 5). Brito and colleagues have recently shown differential activation of white and brown fat in response to fasting, glucoprivation, exposure to a cold environment, as well as a result of activation of the melanocortin 4 receptor, a hypothalamic signaling pathway that partially mediates the actions of leptin [30], [31]. Fasting and cool temperatures induce β3-mediated sympathetic activation of white adipose, resulting in a fall in circulating leptin [32]–[34]. Suppression of leptin then leads to altered hypothalamic activity [35], with the outcome of a much reduced metabolic rate and body temperature. At some point during the torpor bout, arousal is initiated and the SNS activates β3 receptors on brown fat, leading to rapid heat production. Additionally, the SNS activates (or continues to activate) the smooth muscle surrounding peripheral blood vessels, causing vasoconstriction and decreased peripheral blood flow. The generation of heat, and its retention, allow for rapid emergence from torpor, although the timing trigger for torpor recovery is still unknown. Thus, the coordination of sympathetic outflow to these two adipose tissues helps orchestrate the metabolic response to and ultimately survival from acute food shortages.
